# Changes in Renal Function in Elderly Patients Following Intravenous Iodinated Contrast Administration: A Retrospective Study

**DOI:** 10.1155/2014/459583

**Published:** 2014-03-24

**Authors:** Ali Alsafi, Zaid Alsafi, Amish Lakhani, Nicola H. Strickland

**Affiliations:** Radiology Department, Imperial College Healthcare NHS, Hammersmith Hospital, Du Cane Road, London W12 0HS, UK

## Abstract

*Background.* Contrast-induced nephropathy (CIN) is a recognised complication of intravascular administration of iodinated contrast media (ICM). Previous studies suggest a higher incidence in the elderly, but no large study has assessed this to date. We set out to assess changes in creatinine in elderly inpatients following computed tomography (CT) examination and compare those who received intravenous contrast to those who did not.* Methods.* Using the Radiology Information System in two teaching hospitals, inpatients over the age of seventy who had a CT examination and a baseline creatinine were identified and their follow-up creatinine levels were analysed. Elderly inpatients who underwent a noncontrast CT over the same period were used as controls.* Results.* 677 elderly inpatients who received ICM were compared with 487 controls. 9.2% of patients who received ICM developed acute kidney injury (AKI) compared to 3.5% of inpatient controls (*P* < 0.0001). Patients with higher baseline eGFR had a higher incidence of post-CT AKI.* Conclusions.* The incidence of post-CT AKI is higher in patients who received IV ICM compared to those who did not; the difference may be partly attributable to contrast-induced nephropathy. This suggests that the incidence of CIN in the elderly may not be as high as previously thought.

## 1. Background

Intravascular administration of iodinated contrast media (ICM) is used in a variety of diagnostic and therapeutic procedures. While ICM are generally safe, only resulting in minor side effects, they can result acute renal impairment known as contrast induced nephropathy (CIN) [1]. The majority of cases of CIN are self-limiting, although occasionally chronic renal failure ensues necessitating long-term renal replacement. Even when transient, CIN is associated with increased length of hospital stay and significant morbidity [1, 2].

Diabetes mellitus, preexisting renal failure, congestive heart failure, and hypotension, dehydration as well as the use of ACE inhibitors, diuretics, and nonsteroidal anti-inflammatory drugs have all been identified as important risk factors for developing CIN [3, 4]. Hydration in high-risk patients remains the most important preventative measure [5, 6].

Many attempts at defining CIN exist, but the most widely accepted definition is a 25% rise in serum creatinine concentration from baseline or an absolute increase by 44 *μ*mol/L over the 48 to 72 hours following ICM administration, once other causes of renal impairment have been excluded [2, 4].

The use of CT in clinical medicine has exponentially increased over the past two decades, including managing elderly patients, who are thought to be particularly susceptible to developing CIN [7–10]. The true incidence of CIN in the elderly is not known, as most studies addressing the issue assumed that acute kidney injury (AKI) following ICM administration is in fact CIN [11–13]. The results of these studies are therefore likely to be an overestimate, as they failed to compare the incidence of AKI in their patient population to that of a control group. AKI itself is difficult to define, but a practical definition is that of a rapid deterioration in renal function resulting in the inability to maintain fluid, electrolyte, or acid-base homeostasis [14].

CIN has come recently under question as a clinical entity, with suggestions that its incidence and significance have been grossly overestimated. One study even questions the existence of CIN, particularly in patients with preexisting normal renal function [15–17].

In this study, we sought to retrospectively evaluate changes in renal function in elderly patients who underwent a contrast enhanced CT examination and compare to controls who had a noncontrast CT. The aim was to estimate the incidence of post-CT AKI in the elderly.

## 2. Methods

Using the Radiology Information System (RIS) in two teaching hospitals, inpatients over the age of seventy who had a contrast enhanced computed tomography (CT) examination between August 2011 and August 2012 were identified. Demographic and examination data were recorded. Using the hospitals' pathology results systems, the most recent creatinine (Cr) values prior to the contrast examination were recorded. Follow-up Cr values 48–72 hours following the examination were also noted. The most recent Cr prior to the CT examination (up to six weeks) was taken as the baseline against which changes in Cr were compared. eGFR was calculated using the modified diet in renal disease (MDRD) formula [18].

Information relating to prehydration, posthydration, and administration of NAC was not readily available and not recorded, although our standard in-hospital practice is to pre- and posthydrate high-risk patients.

We excluded patients who had no baseline creatinine, those who had no blood tests between 48 and 72 hours following the CT examination, and those on haemodialysis, peritoneal dialysis, or hemofiltration. Patients who had a nephrostomy or renal stent placement within 7 days of the study were also excluded. Patients who had a contrast head CT receiving 50 mL of Omnipaque 300 were excluded. This group constitutes a small number of patients with a significantly lower contrast dose to the remainder of the study population.

All elderly inpatients who underwent a noncontrast examination over the same period were used as controls with the same exclusion criteria. These patients were also identified using the RIS system. Those who developed AKI of an order comparable to that seen in CIN, (25% rise in serum creatinine concentration from baseline or an absolute increase by 44 *μ*mol/L) were identified. The incidence of AKI was calculated for the ICM and control group.

Institutional review board approval was obtained and informed consent was waived prior to commencing this retrospective study.

### 2.1. Statistical Analysis

Two-tailed Fisher's exact test was used to compare categorical data, while two-tailed Student's* t*-test was used to compare numerical data. *P* values <0.05 were considered significant. Odds ratio was calculated using GraphPad Prism 5.0 for Mac OS X (GraphPad Software Inc., San Diego, CA, USA).

## 3. Results and Discussion

920 inpatients over the age of 70 had a contrast enhanced CT examination between August 2011 and 2012, including 825 body CT examinations and 95 head CT examinations; the latter were excluded. A further 81 patients were excluded because they were receiving renal replacement therapy and one patient was excluded because of a recent nephrostomy.

677 patients had a baseline creatinine and at least one further creatinine measurement 48–72 hours following their CT. None of the patients had more than one contrast enhanced examination during the study period. Over the same period, 850 inpatients underwent a CT examination without contrast. 363 of these patients were excluded: 17 because they were receiving renal replacement, 48 who had no baseline creatinine, one who had acute kidney injury predating the CT examination, and 297 who did not have a creatinine level measured 48–72 hours following their CT examination. The remaining 487 cases were used as controls. There was no significant difference in sex or baseline eGFR between the two groups, although the control group was slightly older than the ICM group. (Demographics and baseline eGFR are shown in Tables 1 and 2 resp.)

**Figure 1:**
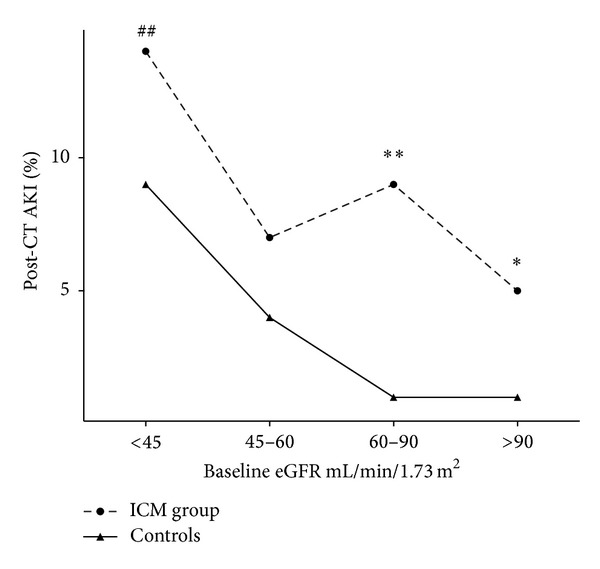
Percentage of patients developing post-CT AKI in the ICM group compared to controls. Using Fisher's exact test: *P < 0.05 and **P < 0.001, comparing the ICM group versus controls and ^##^ *P* < 0.005 comparing to patients with a baseline GFR > 90 mL/min/1.73 m^2^.

In the 48 to 72 hours following intravenous administration of 80–120 mL of Omnipaque 300, 5.9% (13/220) of patients with a baseline eGFR > 90 mL/min/1.73 m^2^ developed AKI compared with 9.1% (19/208), 7.4% (7/95), and 14.9% (23/154) of those with eGFR 60–90 mL/min/1.73 m^2^, 45–60 mL/min/1.73 m^2^, and <45 mL/min/1.73 m^2^, respectively (Figure 1). The relative risk for developing post-CT AKI in patients who had 80–120 mL of ICM with a baseline eGFR < 45 mL/min/1.73 m^2^ compared to those with an eGFR > 90 mL/min/1.73 m^2^ was 1.65 (95% CI 1.25 to 2.18, *P* = 0.0043).

The relative risk for developing post-CT AKI in inpatients who had 80–120 mL of ICM compared with controls is 1.35 (95% CI 1.22 to 1.57, *P* < 0.0001).

Intravenous contrast media (ICM) are widely used in diagnostic and interventional radiology; their use, however, is associated with up to 11% of hospital-acquired AKI [5]. CIN is a cause of significant morbidity and mortality in hospital patients and is thought to be an important cause of AKI in this population alongside sepsis, hypoperfusion, major surgery, and nephrotoxic medication [5, 11–13].

Increasing age is an independent predictor for developing CIN with a fivefold increase in risk in patients over the age of seventy [7].

Previous studies, including MacDonald et al's recent large meta-analysis, showed no difference in incidence of post-CT AKI, between contrast and control groups. These studies assessed significantly younger patient populations than ours (median patient age of 62 versus 82 in our study) and their results may not be generalizable to an older age group [15, 16, 19].

Davenport et al. on the other hand demonstrated that IV ICM is an important risk factor, albeit not the only one, in developing post-CT AKI in an adult patient population [17]. Our results extend these conclusions to a significantly older population, which may be even more susceptible to CIN.

Two studies estimated the incidence of CIN in the elderly at 6% and 14%, respectively. Neither study accounted for other causes of AKI nor did they compare with controls [9, 10]. To our knowledge, this is the first study to compare the incidence of post-CT AKI in elderly patients who received IV ICM with controls who had nonenhanced examinations.

While the excess AKI in the ICM group is, at least partly, likely attributable to contrast administration, there may be inherent differences between the two groups, with patients not receiving ICM being perceived as high-risk and therefore undergoing noncontrast examinations. The fact that the average baseline eGFR, age, and gender distribution are similar in the two groups does not support this.

Furthermore, a significant number of the noncontrast studies were head CT examinations (87%), suggesting that the disease processes for which the two groups presented to hospital may be different, with the ICM group being more likely to have systemic, rather than neurological, symptoms and therefore more likely to develop AKI. This is merely speculative but may partly account for the differences seen in conjunction with true CIN. If this is the case, the incidence of CIN may even be lower.

## 4. Limitations

Although we did not assess for risk factors other than preexisting renal impairment, by using a control population with similar demographics who were inpatients during the same study period, the difference in incidence of AKI may be partly attributable to the administration of IV ICM.

Our results apply to an elderly hospital inpatient population and may not be generalizable to a healthier outpatient population, whose risk of CIN is perhaps even lower.

## 5. Conclusions

The incidence of AKI in our elderly inpatient population was 9.2% after administration of 80–120 mL of intravenous ICM compared with 3.5% in inpatient controls. The difference between the two groups may be, at least partly, attributable to ICM. Caution, however, needs to be exercised in elderly patients with low baseline eGFR (<45 mL/min/1.73 m^2^) as post-CT AKI approaches 15% in this group.

## Figures and Tables

**Table 1:** Demographic data and baseline eGFR (mL/min/1.73 m^2^).

	Body CT (*n* = 677)	Controls (*n* = 487)	*P* value
Average age (range)	80.4 (70–102)	82 (70–103)	<0.0001*
Males (%)	340 (50.2%)	240 (49.3%)	0.7666^#^
Females (%)	337 (49.8%)	247 (50.7%)	
Baseline eGFR > 90	220 (32%)	153 (31.4%)	0.7030^#^

*Unpaired Student's *t*-test; ^#^Fisher's exact test.

**Table 2:** Number of patients developing post-CT AKI in the ICM and control groups with varying baseline eGFR. (eGFR in mL/min/1.73 m^2^).

Baseline eGFR	ICM group (%)	Controls (%)
>90	13 (5.91%) (*n* = 220)	2 (1.31%) (*n* = 153)
60–90	19 (9.13%) (*n* = 208)	2 (1.23%) (*n* = 162)
45–60	7 (7.37%) (*n* = 95)	3 (4.48%) (*n* = 67)
<45	23 (14.9%) (*n* = 154)	10 (9.52%) (*n* = 105)

Total	62 (9.16%) (*n* = 677)	17 (3.49%) (*n* = 487)

## Abbreviations

CIN: Contrast induced nephropathy
ICM: Iodinated contrast media
CT: Computed tomography
AKI: Acute kidney injury
eGFR: estimated glomerular filtration rate
Cr: Creatinine
NAC: n-Acetylcysteine
MDRD: Modified diet in renal disease
RIS: The Radiology Information System.
